# Are Air Pollution, Economic and Non-Economic Factors Associated with Per Capita Health Expenditures? Evidence from Emerging Economies

**DOI:** 10.3390/ijerph16111967

**Published:** 2019-06-03

**Authors:** Muhammad Usman, Zhiqiang Ma, Muhammad Wasif Zafar, Abdul Haseeb, Rana Umair Ashraf

**Affiliations:** 1 School of Management, Jiangsu University, Zhenjiang 212013, Jiangsu, China; mzq@ujs.edu.cn; 2 School of Management and Economics, Beijing Institute of Technology, Beijing 100081, China; wasif.zafar6@yahoo.com (M.W.Z.); abdulhaseeb5288@outlook.com (A.H.); 3 School of Computer Science and Communication Engineering, Jiangsu University, Zhenjiang 212013, Jiangsu, China; ranaumairashraf2002@hotmail.com; 4 Department of Management Sciences, COMSATS University Islamabad, Vehari Campus, Vehari, Punjab 61100, Pakistan

**Keywords:** government health expenditures, private health expenditures, CO_2_ emissions, environment index, economic growth, emerging economies

## Abstract

Environmental pollution, rapid economic growth, and other social factors have adverse effects on public health, which have consequently increased the burden of health expenditures during the last two decades. This paper provides a comprehensive analysis of carbon dioxide (CO_2_) emissions and the environment index, as well as economic and non-economic factors such as Gross Domestic Product (GDP) growth, foreign direct investment, population aging, and secondary education impacts on per capita government and private health expenditures in 13 emerging economies for the time period of 1994–2017. We employ robust econometric techniques in this endeavor of panel data analysis to account for the issues of heterogeneity and cross-sectional dependence. This study applies the Lagrange Multiplier (LM) bootstrap approach to investigate the presence of panel cointegration and empirical results underscore the existence of cointegration among variables. For the execution of long-run analysis, we incorporate the two latest estimators, i.e., continuously updated-fully modified (CUP-FM) and continuously updated- bias corrected (CUP-BC). Findings of long-run elasticities have documented that the air-pollution indicators, i.e., CO_2_ emissions and the environment index, have a positive and significant influence on government health expenditures, while in contrast, both factors negatively influence private health expenditures in emerging economies. We find that economic factors such as GDP growth consistently show a positive impact on both government and private health expenditures, whereas, foreign direct investment exhibits a significant negative and positive impact on government and private health expenditures respectively. Findings of non-economic factors can be used to argue that population aging increases health expenditures while secondary education lowers private health spending in emerging markets. Furthermore, empirical analysis of heterogeneous causality indicates that CO_2_ emissions, the environment index, GDP growth, foreign direct investment, and secondary education have a unidirectional causal relationship with government and private health expenditures. Population aging has a strong relationship of bidirectional causality with government health expenditures and unidirectional causal relationship with private health expenditures. Findings of this paper put forward key suggestions for policy makers which can be used as valuable instruments for better understanding and aiming to maximize public healthcare and environmental quality gains which are highly connected with sustainable GDP growth and developments in emerging economies.

## 1. Introduction

The rapid increase of health expenditures due to widespread environmental deterioration, extensive economic activities, and other social factors is a great challenge for governments and public healthcare policymakers in emerging economies. The main environmental and social cost is linked to air pollution [1]. Air pollution mainly comprises greenhouse gases (GHGs), such as carbon dioxide (CO_2_), sulphur dioxide (SO_2_), and nitrogen oxides (NO_x_) from power plants, manufacturing industries, and vehicles, which generate PM_2.5_ (atmospheric particulate matter (PM) with a diameter of less than 2.5 micrometers). Apergis et al. [2] have argued that on a continuous basis, the accelerated expansion and emission of severe types of GHGs are causing environmental deterioration and threating public health. 

Since 1990, a trend of rapid economic growth has been recorded by emerging economies to satisfy the growing expectations and demands of their rising concentrated populations. As a consequence of this fact the emerging economies and most notably China and India became major CO_2_, SO_2_, and NO_x_ emitter countries. Immense air pollution due to high PM_2.5_ concentrations is estimated to have has caused the annual death rate of 1.2 million people in China and India compared to a rate of 0.1 million in the USA (according to the World Health Organization (WHO), in Bangalore, a 34% increase of air pollutants was recorded within the time period from 2002 to 2010 and in January 2013 in Beijing, the haze caused 98% of health-related economic losses, with an estimated cost of 23 billion RMB (3.7 billion US dollars)) [3].

Moreover, the current statistics of the World Health Organization [3] have noted that the massive increase in air pollution trend will lead to increased premature mortality caused by environmental degradation by 2050. Health damages caused by adverse air quality were valued to represent around 4% of the Gross Domestic Product (GDP) among the 15 countries which had the highest emissions of GHGs in 2012.

According to epidemiological studies, the cases of morbidity and mortality caused by air pollution have increased around the globe during the last decades [4,5]. Policy makers and environmental experts have focused on researching issues related to social and economic conditions which are caused by man-made pollution [6]. The main cause of environmental degradation and a dangerous threat to the quality of a healthy life is greenhouse gas emissions. The high increased levels of GHG emissions have created an alarming situation for the ecosystem and global warming [7].

More recently, the Global Alliance on Health and Pollution (GAHP) declared that unprecedented attention on public healthcare that could be impacted by environment pollution has increased [8]. The World Health Organization (WHO) documented that, in 2016, the worldwide number of premature deaths attributable to ambient (outdoor) air pollution rate was 4.2 million [3]. The WHO recorded that 91% of the global population is living in areas where the WHO air quality standards and guidelines are not met. Moreover, WHO data stated that dangerous levels of air pollution are continuously increasing around the world and nine people out of 10 are breathing air which contains harmful levels of various forms of pollution.

The different harmful effects of air pollution on public health have led to a massive increase of healthcare spending and decrease of labor productivity, which in turn have resulted in an immense social cost burden and generated greater economic pressure [9,10,11]. Economists and policymakers have considered that the social cost aggravated by a higher level of air pollution is one of the great obstacles in the way of long-term economic development. Jacobson [12] showed that the numbers of patients’ hospitalization rates, as well as death rates, are increased by air pollution, which adversely affects labor productivity, industrial production, and growth of the economy. Considerable adverse effects of air pollution on human health have been recorded in the past decades, and these environmental shocks, particularly in emerging economies, have caused enormous damage to public health and increased healthcare expenditures.

The Energy Information Administration [13] reported that in emerging economies, the CO_2_ emissions level share increased from 34.6% to 49.7% within the time span of 1991–2012 compared to the CO_2_ emissions levels around the world. Thus, facing this alarming situation of environmental degradation, it is crucially important to investigate the nexus between GDP growth, air pollution, and government and private health expenditures in emerging economies.

Currently, emerging economies are facing a dual-sword challenging situation where on the one hand enormous economic growth is required and the other hand, they must deal with public health issues and the increasing trends of healthcare expenditures due to the resulting from air pollution and environmental degradation. Unfortunately, growing economies in emerging markets cannot sacrifice intensive economic growth for environmental quality, yet prior research has conclusively proven that economic growth is one of the leading factors that are positively and significantly associated with air pollution [14,15,16].

In the context of public health issues and sustainable economic growth, it is important to estimate the various adverse impacts of air pollution and accurately develop strict regulations for effective environmental quality. Overregulation due to the overestimation of the adverse effects of air pollution could hamper economic growth, while an underestimation of the harmful effects of air pollution could lead to unnecessary substantial losses of economic welfare and the public being unprotected against chronic diseases [17]. Therefore, it has been proposed that it is very critical for economists and policymakers to evaluate the actual level of social costs associated with air pollution because both over and underestimation could impact the economy and public health.

A major cause of non-communicable diseases is air pollution, which must be controlled to a greater extent [18]. Furthermore, in emerging and developing economies, the public response to ambient air pollution has increased pressure on government policies and budgets to maintain the ecosystem, upgrade the public healthcare system, and protect the economy from uncountable losses. Therefore, quantifying the increase in government and private health expenditures caused by air pollution is a crucial component in the policymaking debate.

A comprehensive survey related to empirical studies published during the time period 2006–2015 shows that the regional efficiency of energy and carbon emissions in China remained stable during 1996–2000 in the 9th five-year plan, during the 2000–2005 time period it decreased in the 10th five-year plan, and it showed an increased trend in the 11th five-year plan during 2006–2010 [19]. In China, the SO_2_ shadow price decreases with the increasing GDP per capita, but the intensity of capital labor has a positive and significant effect on the cost of SO_2_ abatement [20]. A systematic review and meta-analysis of pulp and paper industries showed that the main culprit of GHG emissions is the extensive consumption of energy, as 62% of energy is used in the pulp and paper making process, which is responsible for 45% of GHG emissions [21].

Zeng, et al. [22] suggested that government policy makers can control carbon emissions and the price of carbon emission allowances in Beijing through the price of coal, natural gas, and crude oil at a macroeconomic level. Li, et al. [23] investigated the higher energy consumptions and CO_2_ emissions of the nonferrous metals industry (NMI) sector. They argued that the Chinese government should design strict CO_2_ reduction policies for the NMI, to help China attain its ambitious CO_2_ emission reduction goals.

Emerging economies, including China, India, Indonesia, and Brazil, are considered the home of half the world’s total population, but healthcare spending is half of that reported for developed countries. Continuous environmental degradation, extensive economic growth, and a rapid increase of the graying population in emerging economies will create a dangerous and challenging situation for public and private healthcare expenditures in the next ten years. According to the UN, the plus 65-years demographic of the emerging economies will rise to 15% from 10% of the population by 2030.

**Figure 1:**
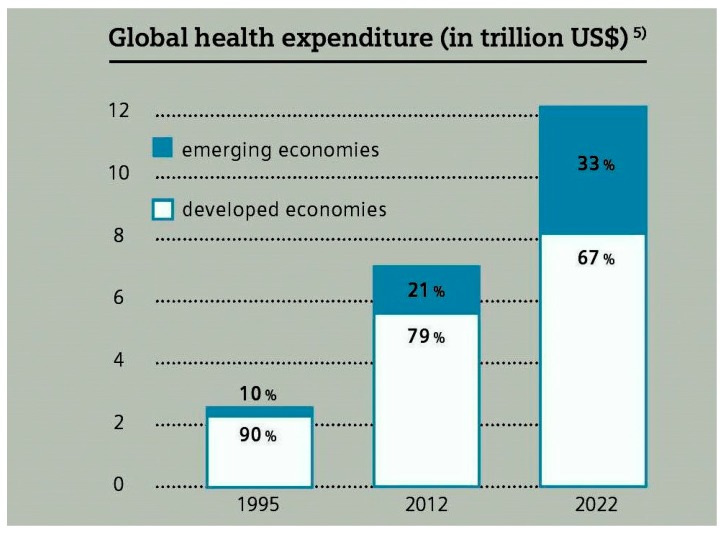
Change in Health Expenditures Globally (in Trillion US$). Source: World Economic Forum.

The “World Economic Forum” reported that an estimation of 1/3 of the global healthcare spending will take place in emerging economies till the end of 2022 [24]. This globally estimated change in health expenditures is shown in Figure 1. Therefore, in this study, we have focused on emerging markets. A total of 13 emerging economies are included in the sample of this study, which covers the five major countries so-called Brazil, Russia, India, China and South Africa (BRICS). Moreover, China, India, and Russia are included in the list of countries that are the top emitters of CO_2_ around the world.

The Frontier Strategy Group (FSG) and Ducker World-wide Healthcare Practice have reported that although there is a deceleration of economic development in China, there is an anticipation of marginal acceleration in overall healthcare expenditures, including the growth of public expenditures by more than 8.4% and private expenditures by more than 8.8%. The acceleration in private healthcare expenditures outpacing public healthcare expenditures clearly indicates that the government is continuously pushing the burden of healthcare budget financing towards the general public [25].

Yang and Zhang [26] have revealed that a 1% yearly increase in PM_2.5_ exposure causes a 2.94% increase in health expenditures of a household. Their estimations suggested that Environmental Protection 13th Five-Year Plan and Ecological (the 13th FYP) could help to reduce national health spending by 47.36 billion U.S. dollars, which is 0.64% of China’s GDP.

This study provides six main contributions to the environmental quality, GDP growth, and health expenditures literature. First, only a few prior studies have focused on the influence of air pollution on government and private health expenditures, so this study comprehensively examines the impact of air pollution on government and private health spending. Discussion and empirical analysis of public healthcare expenditures and the quality of the environment is an emerging research area. Recently, research on the topic of the environment and health has become very important due to the widespread nature of many infectious diseases caused by higher temperatures due to global warming and air pollution [27].

Second, this paper is focused on emerging economies because the environmental degradation and the need for extensive GDP growth represent a dual edged challenge for governments and private healthcare spending in emerging economies which has been ignored in prior research.

Third, to the best of our knowledge, this study is the first to use the environment index, which combines CO_2_, SO_2_, and NO_x_ as air pollution indicators. An investigation of a composite index of different substances of air pollution such as CO_2_, SO_2_, and NO_x_ is critical because various health events have been found to be highly associated with these air pollution factors [28].

Fourth, economic factors such as GDP growth and foreign direct investment (FDI), as well as non-economic variables such as population aging and secondary education are also included as explanatory variables to examine the broader variations in government and private health expenditures. In prior research, the impact of foreign direct investment and secondary education on health spending was rarely examined. Thus, this study empirically explores the impact of all these factors on government and private health spending.

Fifth, the latest data analysis techniques have been employed such as panel cointegration analysis, where we have used the Lagrange Multiplier (LM) bootstrap test which helps to account for problems of heterogeneity and cross-sectional dependence.

Sixth, this study calculates the long-run elasticities and checks the causal relationship among variables by using advanced estimators, including the continuously updated-fully modified (CUP-FM), continuously updated- bias corrected (CUP-BC) and Dumitrescu Hurlin Granger causality test, respectively.

The rest of this paper is organized as follows: in the second section, the related prior literature is explained. In the third section, the data collection, variable specifications, and complete econometric methodology are presented. In the fourth section, the results and findings of our empirical data analysis are given. In the fifth and final section, related discussions and the conclusions are summarized.

## 2. Theoretical Perspective

A wide strand of growing literature and empirical studies has focused on the nexus between environmental quality, economic growth, and health expenditures. For instance, Micheal, et al. [29] aimed to analyze the relationships between CO_2_ emissions, economic growth, and health expenditures. They used Fully Modified Ordinary Least Squares (FMOLS) as an econometric methodology for long-run analysis to examine the relationship between variables over the time span of 1970–2008 in Ghana. They concluded that GDP and health expenditures are positively and significantly associated, while no relation was found between CO_2_ and health expenditures.

Chaabouni, et al. [30] examined the linkage between CO_2_ emissions, GDP growth, and health spending in 51 countries over the time period of 1993–2013. They employed a dynamic simultaneous-equation model and generalized method of moments (GMM) to examine the relationship between these three factors. Their study results revealed that both CO_2_ emissions and GDP growth have a positive and significant effect on health spending. More recently, Saida and Kais [31] applied the Autoregressive Distributed Lag (ARDL) method to Sub-Saharan African countries for the time span 1990–2015, and argued that an increase of 1% GDP growth and 1% CO_2_ emissions could cause a 0.332% increase and 0.066% decrease in health spending, respectively.

However, most prior studies have investigated the relationship between only two variables. Since 1960, several studies have examined the first-line nexus between the growth of GDP and healthcare spending, but the results of those studies are still inconclusive. However, a large number of researchers have reached the consensus that an increase in health spending occurs due to economic growth [32,33,34,35]. Most of the empirical research has relied on time-based econometric methods to investigate the relationship between the growth of GDP and healthcare expenditures [36,37,38,39]. Moreover, Clemente, et al. [40] incorporated the cointegration techniques for a short- and long-term analysis of the relationship between these two factors of economic growth and spending of healthcare. Additionally, some researchers have focused on examining the various effects due to structural changes and reported dual relationship between these two variables of healthcare expenditures and growth of GDP [41,42].

In the second tier of prior studies, the nexus between environmental quality and health expenditures has been tested. For instance, Jerrett et al. [1] found that there is a positive association between air pollution and health expenditures. Further, Mehrara, et al. [43] investigated the relationship between environmental quality and health spending in 114 developing countries over the time span of 1995–2007. They employed cointegration techniques for panel data and found a direct connection between the environmental quality and spending of healthcare in the short-run, as well as in the long-run. However, complete systematic analyses examining the nexus between the three variables of environmental health, GDP growth, and health expenditures are scarce.

The coefficients of air pollution in the short-term, as well as in the long-term, have verified that the phenomenon of air pollution has long-term effects on public healthcare because it is the major cause of continuous environmental degradation and the main factor behind chronic diseases [44,45]. Air pollution due to the inclusion of ozone, carbon dioxide, particulate matters, nitrogen oxide, and sulphur dioxide causes various physical changes in the body and clearly causes the symptoms of different diseases which are threatening to human health. According to a report published by the World Health Organization [46], outdoor and indoor air pollutions are listed as the 14th and 10th leading risk factors of deaths globally, respectively.

Raeissi, et al. [47] found that health spending is positively affected by air pollution in the long-run, as a 1.00% increase in CO_2_ emissions could lead to a 1.16% and 3.32% increase in private and government health spending, respectively. They concluded that in the long-term, air pollution has a strong impact on health spending compared to the short-term. In the last two decades, expenditures of public and private healthcare systems have faced disputes, prominently becoming a heated issue during the global economic recession, which placed many constraints on government budgets and shifted the burden to private health expenditures. The International Monetary Fund (IMF) has given recommendations that provisions of loan conditions for private healthcare systems should be increased by countries [48].

The debate on public and private healthcare systems is divided into two groups: one is those looking for widespread public healthcare availability and the other represents those supporting a private sector for healthcare where the government has typically failed to provide public healthcare. Advocators of a private healthcare system have pointed to the evidence that private clinics are more preferable to different impoverished patients [49]. Further, studies have suggested that the responsiveness and efficiency are high in the private healthcare sector due to market competition, and those studies have also indicated that the private sector helps to overcome the inefficiency and corruption issues of government public healthcare [50]. In contrast, advocators of the public healthcare system have highlighted the issues of inequities and the inability of the poor general public to access and pay for healthcare services provided by the private sector.

Economic policies of different countries have an effect on the healthcare system and health inequalities [51]. Currently emerging economies are facing deficit budgets in different public sectors, and this macroeconomic pressure creates high frictions in the financing of healthcare expenditures which are increasing over time and spill over the public healthcare budget. Private insurance and out-of-pocket payments are considered alternative approaches for reducing the impact on public financing. Although there are differences between public and private healthcare spending amongst emerging market countries, in broader patterns, there is interconnectivity among the status of public health and healthcare expenditures. Environmental pollution and older-age populations in emerging economies considerably increase health expenditures because air pollution harmfully impacts elderly people and they often require costly medical facilities and treatments for chronic illnesses and multiple morbidities.

Spix and Wichmann [28] showed that health events are significantly associated with different air pollutants such as carbon dioxide, sulphur dioxide, and nitrogen oxide. Nitrogen oxide supports the materialization of ozone and acid rain. Air containing a large amount of nitrogen oxides can cause respiratory problems, especially in the elderly, children, and asthmatic patients. Sulphur dioxide emanations are mainly from the burning of coal and oil. A high concentration of sulphur dioxide in ambient air can lead to throat and nose burning, resulting in breathing difficulties.

Although, some research has examined the topic of CO_2_, SO_2_, and NO_x_ emissions’ impact on health spending, their findings are inconclusive. For instance, Narayan and Narayan [52] investigated the association between the quality of the environment and healthcare expenditures in eight countries of the Organization for Economic Co-operation and Development (OECD). Their study findings showed that income, CO_2_ emissions, and SO_2_ emissions are positively associated health expenditures, while no impact was found for NO_x_ emissions. Moreover recently, Saida and Kais [31] applied ARDL estimation to panel data of Sub-Saharan African countries to analyze the nexus between CO_2_ emissions, NO_x_ emissions, GDP growth, and health expenditures for the time period 1990–2015. They found a positive impact of economic growth on health spending, while both CO_2_ and NO_x_ emissions negatively influenced the health expenditures in the long-run.

There are extensive harmful impacts of air pollution on environmental quality and human health, particularly wide-ranging effects in the case of emerging economies and developing countries such as China [53], South Africa, Brazil, Russia and India. An increased trend of healthcare spending and immense reduction of labor productivity have been recorded due to the harmful impacts of air pollution on public health, which have ultimately caused massive social costs and exerted pressure on growth of the economy [9,10,11]. Literature related to foreign direct investment (FDI) has revealed various impacts on the economy and social spending. Much prior research has considered FDI as a determinant of social spending, e.g., [54,55,56]. Conversely, only a few studies have determined the relationship between FDI and health [57]. For instance, Huber, et al. [58] specifically found a positive impact of FDI on health spending.

Population aging has been considered an important determinant of health spending in previous studies, and empirical findings of those studies have reported that population aging (over the age of 65) significantly and positively impacts health expenditures, e.g., [33,59,60]. Furthermore, the growing current literature and empirical studies have started focusing on the relationship between education level and public health.

For example, Lu, et al. [61] proposed that conditions of public health depend on GDP per capita, the quality of the environment, education level, and medical facilities. Their empirical results have indicated that education level (average years of education) significantly reduced the total mortality rate. The public basic education level is necessary to develop more maturity for the proper treatment of illness and more sensibility to protect the population from chronicle diseases caused by environmental pollution. Thus, the education level could significantly impact healthcare expenditures.

## 3. Research Methodology

### 3.1. Data

This empirical study has focused on emerging economies by following the Morgan Stanley Capital International (MSCI) market classification of emerging economies. Therefore, for a completely balanced panel data set, we have collected the data of 13 emerging economies for the time period of 1994–2017. The sample covers the countries Brazil, Chile, China, Colombia, Egypt, India, Indonesia, Malaysia, Peru, Russia, South Africa, Thailand, and the Philippines. The selection of countries and time period is purely based on the availability of data. All variables’ names, acronyms, data sources, and complete descriptions are given in Table 1.

### 3.2. Econometric Modeling

Several prior studies have proposed the effect of environmental degradation, such as effects of CO_2_ emissions and NO_x_ emissions on general health expenditures [1,30,31,52,62,63]. Moreover, some prior studies have also tested the impact of economic growth on expenditures of healthcare [64,65,66]. To follow, the purpose of this study is to investigate the nexus between environmental pollution, economic growth, and health expenditures by using a data set of 13 emerging economies for the time period of 1994–2017. We have employed the following four models based on the prior literature as discussed earlier in this study:

Model 1: GHE = f (COE, GDP, FDI, PAG, EDU)
Model 2: GHE = f (ENVI, GDP, FDI, PAG, EDU)
Model 3: PHE = f (COE, GDP, FDI, PAG, EDU)
Model 4: PHE = f (ENVI, GDP, FDI, PAG, EDU)

Here, the first and second models are related to government health expenditures, while the third and fourth models are related to private health expenditures as a function of environmental pollution factors, economic factors, and non-economic factors. 

In this study, we have incorporated the air pollution effects in the context of carbon dioxide emissions and a composite of the environment index which includes three main air pollution indicators, CO_2_, SO_2_, and NO_x_ emissions. All variables’ data is expressed in different measurement units, such as per capita, percentages, and U.S. dollars. Therefore, before analysis of any aggregation, the data should be in a normal form. For data normalization, all key variables of this study are converted into the values of natural log forms. Natural logarithms of all variables smoothed the whole data for analysis. Dynamic properties of a data set can be ignored by using the natural logarithmic of data transformation [67]. A log-linear form of a data set provides more efficient and consistent results compared to a data set of a simple-linear form [68]. Moreover, if the coefficients are estimated by variables which have been transformed into natural-log form, then the results can be interpreted in point of elasticity terms. The natural-log transformations of four models of this study in the shape of econometric equations are given as follows:

Model 1: lnGHEit = β0 + β1lnCOEit + β2lnGDPit + β3lnFDIit + β4lnPAGit + β5lnEDUit + εit(1)

Model 2: lnGHEit = δ0 + δ1lnENVIit + δ2lnGDPit + δ3lnFDIit + δ4lnPAGit + δ5lnEDUit + εit(2)

Model 3: lnPHEit = γ0 + γ1lnCOEit + γ2lnGDPit + γ3lnFDIit + γ4lnPAGit + γ5lnEDUit + εit(3)

Model 4: lnPHEit = ∅0+∅1lnENVIit + ∅2lnGDPit + ∅3lnFDIit + ∅4lnPAGit + ∅5lnEDUit + εit(4)

Here, lnGHE is the log of government health expenditures, lnPHE is the private health expenditures, lnCOE represents the natural log of carbon dioxide emissions, lnGDP shows the natural log of economic growth, lnFDI is the log of foreign direct investment, lnPAG represents the log of population aging, and lnEDU shows the log of education. The β, δ, γ, and ∅ are the slope intercepts of the first, second, third, and fourth models of this study, respectively. The subscripts i and t represent the number of countries (1, 2, 3 ….N) and the time period (1994 to 2017), respectively. The error term of each econometric model of this study is indicated by ε_it_. 

### 3.3. Methodological Framework

This study follows the complete package of the following methodological framework.

#### 3.3.1. Cross-Sectional Dependence Test

The empirical literature has suggested that variables related to time series possess various properties, such as those that are stationarity or non-stationarity. These properties can be tested by first- or second generation tests of the unit root, which can be selected on the basis of a cross-section independence assumption. Generally, in the case of panel data, the variables of different countries are associated with each other due to the regional and global inter-linkage between those countries. If studies neglected to a incorporate cross-section independence assumption, then it could lead to misappropriate estimations [69]. Therefore, we have incorporated the Lagrange Multiplier (LM) test which was suggested by Breusch and Pagan [70]. The LM test for cross-sectional dependence is examined by using the following equation:

(5) $$y_{it}=\alpha_{i}+\beta_{i}x_{it}+\mu_{it}$$

Here, *i* and *t* indicate the dimensions of cross-sections and time period of this study, respectively. These tests have null and alternative hypotheses about cross-sectional independence and cross-sectional dependence, respectively.

Furthermore, for the investigation of cross-sectional dependence, we have employed the cross-sectional dependence (CD) test which was proposed by Pesaran [71]. The following equation is used for an inspection of cross-sectional dependence by the CD test:

(6) $$CD=\sqrt{\frac{2T}{N(N-1)}}\left(\sum_{i=1}^{N-1}\sum_{k=i+1}^{N}\rho_{ik}\right)$$

Here, the sample size is shown by *N*, the time period of this study is indicated by *T,* and correlations among errors of different cross-sections of country *i* and *k* are presented by $\rho_{ik}$.

#### 3.3.2. Panel Unit Root Test

The results of the tests used in this study for an investigation of cross-sectional dependence have provided evidence of the existence of dependence among cross-sections. Therefore, for examining the residual stationarity, we have used second-generation panel unit root tests to deal with cross-sectional dependence because panel unit root tests of first-generation could provide ineffective estimations under the existence of cross-sectional dependence [72]. This study applied two approaches for testing panel unit roots: first, cross-sectionally augmented IPS (CIPS) and second, cross-sectionally augmented ADF (CADF). The following 7th and 8th equations can be employed for testing CIPS and CADF unit roots tests which were introduced by [73]:

(7) $$\Delta Y_{it}=\gamma_{it}+\chi_{i}Y_{i,t-1}+\lambda_{i}T+\sum_{k=1}^{n}\pi_{ik}\Delta Y_{i,t-k}+\mu_{it}$$

Here, the difference operator is indicated by Δ and the main variable of analyses is shown by *I_it_*, while *γ*, *T* and *μ_it_* represent the individual intercept, time trend, and error term, respectively. Furthermore, the second panel unit root test of this study is CADF. The standard “Augmented Dickey-Fuller (ADF)” regression can be formulated by adding averages of cross-sectional lagged levels (${\bar{X}}_{t-1}$) and the first difference values of individual series. The following equation could be used as the CADF test:

(8) $$\Delta X_{it}=\alpha_{i}+\beta_{i}X_{i,t-1}+\delta_{i}{\bar{X}}_{t-1}+\lambda \Delta {\bar{X}}_{t}+\mu_{it}$$

Here, ${\bar{X}}_{t}$ represents the averages at time span *t* of all given *N* observations of the sample and the equation included it as a proxy of unobserved effects by common factors. 

#### 3.3.3. Panel Co-Integration Test

When variables in a particular study are not stationary at different levels, then it is crucial to test cointegration among these variables for statistically accurate and economically meaningful estimations of the coefficient in the long-run analysis. To examine the existence of cointegration among government health expenditures (GHE), private health expenditures (PHE), CO_2_ emissions (COE), the environment index (ENVI), economic growth (GDP), foreign direct investment (FDI), population aging (PAG), and secondary education (EDU), this study applies the LM bootstrap technique for the cointegration test which was developed by [74]. They have suggested the following equation to measure the bootstrap test:

(9) $$\begin{array}{l} y_{it}^{*}={\hat{\alpha }}_{i}+x_{it}^{{*}^{\prime }}{\hat{\beta }}_{i}+z_{it}^{*}\\ with\\ x_{it}^{*}=\sum_{j=1}^{t}\Delta x_{ij}^{*}, \end{array}$$

Here, ${\hat{\alpha }}_{i}$ and ${\hat{\beta }}_{i}$ are estimated from the fully modified forms of *α_i_* and *β_i_*. The null hypothesis of the LM bootstrap test is that panel data displays cointegration among variables. This test works well in the case of a small sample and is generally suitable, allowing both cases (between and within) of the dependence of all cross-sectional units. Furthermore, the LM bootstrap test helps to account for problems of heterogeneity and cross-sectional dependence in the procedure of cointegration estimation among variables. 

#### 3.3.4. Long-Run Estimation Test 

For the estimation of long-run analysis among all dependent and independent variables, we have used two estimators which were suggested by Baia and Kaob [75], and Bai, et al. [76]. They constructed two estimators which can be used by the following equation to account for biasness issues which could arise from serial correlation, cross-sectional dependence, and endogeneity:

(10) $$\left({\hat{\beta }}_{CUP},{\hat{F}}_{CUP})=\arg\min\frac{1}{nT^{2}}\sum_{i=1}^{n}{\left(y_{i}-x_{i}\beta \right)}^{\prime }M_{F}(y_{i}-x_{i}\beta \right)$$

Here, *β* is the estimated coefficient obtained through applying repeated Fully Modified Least Squares (FM-OLS), which uses the residuals of the previous stage until full convergence occurs. The *F* and $M_{F}=I_{T}-T^{-2}FF^{\prime },I_{T}$ explain the common factors that assumed by error terms and identify the matrix dimension *T*, respectively. Hence, the allocation of initial estimators is based on F and it will continue until the complete convergence. The first estimator is known as continuously updated-fully modified (CUP-FM) and the second estimator is named continuously updated- bias corrected (CUP-BC), and both of these estimators are updated continuously till the whole convergence is completed [76]. In the case of exogenous regressors, these estimators provide unbiased and consistent results. Moreover, these estimators also deal with mixed factors of *I(1)/I(0)* and give robust outcomes. Even if endogeneity is absent, these tests can estimate the consistent findings because estimators are based on FM-OLS [76].

#### 3.3.5. Granger Causality Test

Dependent variables that are associated with independent variables can be evaluated by long-run econometric methods but the estimation of the direction of casual association between the concerned variables in the short-run is necessary for policymaking. Therefore, for better understanding the direction of the relationship of government and private health expenditures with air pollution and other determinants, we have employed the latest and advanced form of the simple Granger causality test which was introduced by Dumitrescu and Hurlin [77]. This test has flexible properties because it can be used for unbalanced and heterogeneous panels with *T < N* and *T > N*. A standard regression of Granger causality is incorporated for individually testing of each cross-section. This test allows differences among all coefficients by cross-sections and takes average values of test statistics through all units of cross-sections. A causality test for panel data is normally based on the bivariate model which can be written in the form of the following equation:

(11) $$y_{i,t}=\alpha_{i}+\sum_{k=1}^{k}\lambda_{i}{}^{\left(k\right)}y_{i,t-k}+\sum_{k=1}^{k}\beta_{i}{}^{\left(k\right)}x_{i,t-k}+\varepsilon_{i,t}$$

Here, $\alpha_{i}$ is the slope intercept, the slope coefficients are indicated by $\lambda_{i}$ and $\beta_{i}$, and the lag lengths in numbers are shown by k. 

## 4. Results and Discussion

Before starting the step-by-step empirical analysis, we have calculated the annual average growth rate of all variables. The basic purpose of this calculation is to understand and compare the annual average growth rate of government and private healthcare expenditures among various emerging economies caused by the annual average growth rate of air pollution. Table 2 shows the average values of annual growth rates. The results reveal that the highest annual growth rate of GHE is in China, with a value of 38.4225%, followed by South Africa and Peru, with values of 15.7234% and 13.9482%, respectively. 

The lowest annual growth rate of GHE is in Russia with value 1.1669%, followed by Chile and Thailand, with values of 3.6638% and 3.6945%, respectively. The highest annual growth rate of PHE is in India, with a value of 9.5420%, followed by Colombia and China, with values of 9.4816% and 9.1511%, respectively. Additionally, the lowest annual growth rate of PHE is recorded in countries such as Thailand (0.3262%), the Philippines (2.1023%), and Chile (2.4402%). In terms of environmental degradation, countries that possess the highest annual growth rate of COE are China (4.9233%), Peru (4.3294%), and the Philippines (3.6825%). 

In contrast, countries that possess the lowest annual growth rate of COE are South Africa, Thailand, and Colombia, with values of 0.4066%, 0.9294%, and 1.0592%, respectively. Moreover, in the case of ENVI, the result shows that the three major countries responsible for air pollution, with the highest annual growth rate of ENVI are China, India, and Peru, with values of 4.8792%, 4.1150%, and 4.0912%, respectively. The first step of the empirical analysis of this study is related to examining the issue in the panel data set of cross-sectional dependence. The results of cross-sectional dependence are given in Table 3. We have employed the LM test and CD test as suggested by Breusch and Pagan [70] and Pesaran [71], respectively. The results of these tests report on the presence of cross-sectional dependence. 

In the second step of the empirical analysis after the confirmation of cross-sectional dependence, we have incorporated second-generation unit root tests to investigate the stationarity properties of panel data. We have used the second-generation tests CIPS and CADF which were proposed by Pesaran [73], because these tests account for the issue of cross-sectional dependence existence. The first-generation test can provide ambiguous results because it neglects the issue of cross-sectional dependence existence. The reliable results of CIPS and CADF tests under the presence of cross-sectional dependence are given in Table 4. The results of both of these panel unit root tests showed stationarity which means no unit root in panel data of this study at first difference. 

In the third step of the empirical analysis, panel cointegration among all the variables of this study was tested. Westerlund and Edgerton [74] proposed a test to examine the cointegration, which is named the LM bootstrap test. Therefore, this study incorporates the LM bootstrap technique for testing the cointegration among variables. The results of this test are reported in Table 5. The results indicate the strong presence of cointegration because the null hypothesis about cointegration existence cannot be rejected with a *p*-value near to one in each model. 

Overall, in the four models, we have found that government health expenditures and private health expenditures are cointegrated with CO_2_ emissions, the environment index, economic growth, foreign direct investment, population aging, and secondary school enrollments, supporting the existence of a relationship among these variables in the long-run.

In the fourth step of the empirical analysis, we have analyzed the variables for a long-run relationship. In prior literature of econometric methodologies, there are various methods to examine the elasticities for long-run analysis. We have applied CUP-FM and CUP-BC methodology for long-run analysis in this study, which were introduced by Bai et al. [76]. The results of the long-run panel analysis are presented in Table 6. The results of the first and second models revealed the positive and significant impact of COE and ENVI on GHE at a 1% level of significance. A one percent increase in COE and ENVI will increase the GHE by 0.10–0.15% and 0.04–0.07%, respectively. 

These results imply that environmental pollution through CO_2_ emissions and also by combined emissions of CO_2_, SO_2_, and NO_x_ increases the government budget of healthcare spending and also builds pressure for controlling the environmental degradation among emerging economies. This finding, that CO_2_ emissions has a positive and significant impact on health spending, is consistent with research by Raeissi et al. [47] in Iran; Micheal et al. [29] in Ghana; and Chaabouni et al. [30] in 51 countries.

However, in the case of PHE, we have found different findings. For instance, coefficient values for COE and ENVI showed a negative and significant connection with private health expenditures at 1% and 5% levels of significance. There is a one percent increase in COE and ENVI when PHE decreases by 0.16–0.05% and 0.02–0.02%, respectively. These results infer that in the case of emerging economies, the CO_2_ emissions and emissions of SO_2_ and NO_x_ lower the burden of private healthcare spending. These findings are in contrast to the study by Raeissi et al. [47] in Iran. 

The GDP results of all four models in this long-run analysis showed a unanimously positive association with both government and private health expenditures at a 1% level of significance. A one percent increase in GDP could lead to an increase of 1.73–1.13%, 1.55–1.17% and 1.13–1.00%, 1.22–1.02% of GHE and PHE, respectively. The results of an increased trend of government and private health expenditures with the increase of GDP growth show that if emerging economies damaged the environmental health through vast economic development, then they could face the challenge of higher healthcare expenditures in the repercussions of air pollution. This finding of the relationship between GDP growth and healthcare spending is consistent with prior studies, e.g., [29,78]. 

The other three determinants used in this study, FDI, PAG, and EDU, have different impacts on government and private health spending. First, FDI showed a negative relation with government health expenditures, while it was positively connected with private health expenditures at a 1% level of significance. These findings imply that FDI lowers the government expenditures through providing better working conditions and health services to the workforce of the host country. Additionally, it increases the private health spending because FDI increases the wages and living standards of peoples of the host country so their demand for public healthcare is increased. Our study findings of the increase in private health expenditure due to the factor of FDI are consistent with a prior study [58]. 

Second, PAG consistently showed a positive and significant connection with both government and private health expenditures in all four models of this study. This finding is used to sturdily argue that the population aging factor is a strong determinant of increasing the health expenditures of government and private systems in emerging economies. The findings of the PAG relationship with both GHE and PHE are consistent with an empirical study [79]. However, they are the opposite of findings of Yorulmaz [80], who argued that population aging has no significant impact on healthcare expenses. Third, the secondary education (EDU) variable showed a significant positive and negative relationship with GHE and PHE, respectively, which inferred that secondary school enrollment in emerging economies could increase government health expenditures, while, lowering private health spending.

The fifth step of the empirical analysis determined the causality effects among all the variables considered in this study. An approach of panel causality was employed in this study, which was introduced by Dumitrescu and Hurlin [77]. Table 7 depicts the results of the pair-wise panel causality test. In the three main nexuses of this study, the results showed the causality effect of the unidirectional connection among them. The first nexus between GHE, PHE, and environmental degradation measured as CO_2_ emissions and the environment index indicated the unidirectional causality effect. The results of the unidirectional causal connection among these variables are in line with and support prior research, e.g., [30,43,81]. 

The second nexus of the unidirectional casual association is found among GHE, PHE, and GDP. The findings of these casual relations are consistent with the studies of Balaji [82] in four Indian states, Ayuba [83] in Nigeria and Hartwig [84] in 21 countries of OECD. However, opposed to some studies, such as that by Devlin and Hansen [85], it found no causal relation among health expenditure and GDP growth, and others have found a casual bidirectional connection between GDP growth and spending of healthcare, e.g., Amiri and Ventelou [86] in OECD and Chaabouni and Abednnadher [87] in Tunisia.The third nexus of unidirectional casual connections is found between COE, ENVI, and GDP. This finding is consistent with the study of Mehrara et al. [43], in which they analyzed data of 114 countries for the time span 1995–2007 and reported unidirectional causality among CO_2_ emissions and economic growth. However, this finding is opposed by a few studies, e.g., the empirical results of some studies have claimed bidirectional causality [88,89,90]. 

The remaining variables exhibited different causality trends. For instance, the relationship of FDI exhibits unidirectional causality with both GHE and PHE. However, PAG shows a relationship of bidirectional and unidirectional causality with GHE and PHE, respectively. Further, the results of the analysis indicate that EDU has a unidirectional causal relationship with both GHE and PHE. 

## 5. Conclusions

The difference in the GDP growth among different emerging economies is an economically important factor determining public healthcare. Healthcare is an essential factor of labor productivity to maintain growth in the economy. Many adverse consequences of air pollution on public health which lead to massive increases of healthcare spending and a decrease of labor productivity, cause an immense social cost burden and ultimately exert pressure on economic development [9,10,11].

In the context of air pollution, economic, and non-economic factors, we have investigated the impact of these factors on government and private health expenditures in 13 emerging economies for the time period of 1994–2017. The empirical investigation started from cross-sectional dependence testing. Both the CD test and LM test reported the presence of cross-sectional dependence because the null hypothesis of no cross-sectional dependence was rejected. Furthermore, the results of the second-generation tests CIPS and CADF for panel unit root analysis showed stationarity, which meant that there was no unit root in the panel data of this study at first difference. 

Under the conditions of cross-sectional dependence presence and non-stationarity of the panel at different levels, we have employed the cointegration test of LM bootstrap as suggested by Westerlund and Edgerton [74]. The finding of the cointegration test of LM bootstrap is indicated by the strong presence of cointegration because the null hypothesis of cointegration existence cannot be rejected with a p-value near to one in each model. Next, for the variables’ long-run empirical analysis, CUP-FM and CUP-BC estimation techniques are used and these estimators are updated continuously till the whole convergence is completed [76]. In the case of exogenous regressors, both estimators provide unbiased and consistent results.

In the long-run analysis, the results of the first and second models revealed a positive and significant impact of CO_2_ emissions and the environment index on government health expenditures. However, in the case of private health expenditures, we have found different findings that CO_2_ emissions and the environment index are negatively and significantly connected with private health expenditures. The results of the economic growth impact in all four models of long-run analysis showed a unanimously positive association with both government and private health expenditures. Population aging showed a substantial positive and significant impact on government and private health expenditures. Statistics of the United Nations estimate that by 2030 the countries of emerging economies will face an increase of 15% from 10% in the demographic of the 65-years-old population. Thus, emerging economies face a challenging situation to combat air pollution and population aging factors in concerns of the budget for healthcare expenditures. 

The direction of the causal association between variables in the short-run is necessary for policy making. Thus, an advanced form of the simple Granger causality test is employed in this study, which was suggested by Dumitrescu and Hurlin [77]. The results of the heterogeneous causality test indicated that CO_2_ emissions, the environment index, GDP growth, foreign direct investment, and secondary education have a unidirectional causal relationship with government and private health expenditures. Population aging exhibited a strong relationship of bidirectional causality with GHE, while it showed a unidirectional causal relationship with PHE.

### Policy Implications 

The costs associated with a comprehensive healthcare system are indisputable. The substantial increase in healthcare expenditures showed that the relationship between ecosystem, environmental degradation, and public healthcare policies cause severe pressure on government budgets. Air pollution is the main component of all costs associated with environmental degradation [1,59]. As the World Economic Forum reported, estimated 1/3 of all globe healthcare expenditures will take place in emerging economies by 2022. Since 2012, the major economies in these emerging markets have been accountable for 50% of emissions of CO_2_ globally. This paper provides considerable evidence that CO_2_ emissions and the composite environment index of CO_2_, SO_2_, and NO_x_ emissions significantly increase the government health expenditures. Therefore, the government of emerging economies must take stern actions on environmental quality.

Although the increased level of health spending will burden government budgets, it might help to provide a sense of social security, economies of scale, and efficiency of resource allocation which could lead to stimulated GDP growth [91,92]. Hence, the results of our study suggest that if countries fail to consider issues of environmental quality in designing health policy, they could face the problem of an extra increase in their healthcare spending budgets. Moreover, a continuous increase of healthcare expenditures could impact the budgets of other sectors, such as infrastructure developments, maintaining quality standards of environmental quality, education, and others. This infers that if this issue is not properly tackled, then it could lead to severe pressure on the government because of the less available budget for catering for the increased gradation in the system of environmental quality.

The results of this empirical study provide substantial support to policymakers as a useful instrument for better understanding and aiming to maximize the public healthcare and environment quality gains, which are highly connected with long-run GDP growth and the development of a country. Overall, the findings of this study can be used to show that social well-being can be achieved by an appropriate level of healthcare expenditures and by obtaining a high-quality environment. For the management of environmental quality, healthcare spending, and sustainable economic growth, considerable attention of policymakers and governments is required to endorse strict rules concerning the use of cleaner and environmentally friendly energy consumption sources in emerging economies. 

## Figures and Tables

**Table 1:** Variables and descriptions

Variable Names	Acronym	Description
Dependent variables		
Government health expenditures	GHE	Domestic general government health expenditure per capita
Private health expenditures	PHE	Domestic private health expenditure per capita
Independent variables		
Carbon dioxide emission	COE	CO_2_ emissions in metric tons per capita
Environment index	ENVI	Authors’ calculated
Economic growth	GDP	GDP per capita that was constant in 2010 in US$
Foreign direct investment	FDI	Foreign direct investment, net inflows in % of GDP
Population aging	PAG	Percentage of total population that is 65 years old and above
Education	EDU	Percentage of gross secondary school enrollment

*Notes*: The environment index (ENVI) is measured by combining carbon dioxide (CO_2_), sulphur dioxide (SO_2_), and nitrogen oxide (NO_x_) as the three main air pollution indicators. We have employed principal component analysis (PCA) for these three air pollution indicators to develop a composite environment index. The weighted environment index is created by applying PCA to the annual emissions of GHGs, including CO_2_ emissions per capita, SO_2_ emissions per capita, and NO_x_ emissions per capita for the 13 emerging economy countries. These three GHGs, including CO_2_, SO_2_, and NO_x_ are potentially connected factors that have caused air pollution. Generally, CO_2_ emissions are considered the main culprit of air pollution, but, here, we have used the two other main GHGs’ combined effect with CO_2_ emissions, which will help us compare CO_2_ emissions’ effect and its combined effect with other GHGs on health expenditures. Table 2 shows the annual average growth rate results of ENVI of each country calculated by the PCA. All variables’ annual data used to establish a balanced panel for analysis has been collected from the online data source of World Development Indicators (WDI).

**Table 2:** Annual Average Growth Rate.

Countries	GHE	PHE	COE	ENVI	GDP	FDI	PAG	EDU
Brazil	3.8915	3.8421	2.9578	2.5692	1.5275	19.6882	2.8660	0.2532
Chile	3.6638	2.4402	2.3799	2.3096	3.1102	10.2850	2.1501	1.6115
China	38.4225	9.1511	4.9233	4.8792	8.4938	−5.3414	2.2919	3.5842
Colombia	5.4462	9.4816	1.0592	0.9585	2.3947	16.0033	2.4197	1.9857
Egypt	3.8450	4.8434	1.1311	0.9713	2.1979	−2.4639	0.4314	0.6837
India	5.9420	9.5420	2.1455	4.1150	5.3101	16.5162	1.6342	3.0453
Indonesia	9.4873	8.7025	1.7821	1.6006	3.0625	−2.2174	1.0473	2.8969
Malaysia	13.9085	7.5052	2.7723	2.6087	3.0970	17.0261	2.2143	1.3339
Peru	13.9482	5.6028	4.3294	4.0912	3.1336	25.4681	2.2225	1.1828
Russia	1.1669	5.3764	2.0202	1.8964	2.8900	17.5171	1.8984	0.8039
South Africa	15.7234	4.9103	0.4066	0.4086	2.7859	23.5422	0.5988	0.6958
Thailand	3.6945	0.3262	0.9294	0.8735	1.4925	107.4528	1.5415	1.6456
Philippines	6.1354	2.1023	3.6825	3.5565	2.4656	27.2598	3.3556	8.2848

**Table 3:** Cross-Sectional Dependence Analysis.

Variables	LnGHE	lnPHE	lnCOE	lnENVI	lnGDP	lnFDI	lnPAG	lnEDU
LM-test	538.70 ***	377.60 ***	487.92 ***	471.05 ***	476.15 ***	123.88 ***	1214.09 ***	484.60 ***
*p*-values	0.000	0.000	0.000	0.000	0.000	0.000	0.000	0.000
CD-test	34.40 ***	35.80 ***	24.80 ***	23.85 ***	41.78 ***	3.61 ***	38.29 ***	22.34 ***
*p*-values	0.000	0.000	0.000	0.000	0.000	0.000	0.000	0.000

Note: *** statistical significance at 1% level. Reported results are generated through Pesaran [71] CD test, in this test the independence of cross-sections is null-hypothesis and statistical distribution is followed the norms of two-tailed standard. Here, Lagrange Multiplier (LM) test, cross-sectional dependence (CD) test, lnGHE is the log of government health expenditures, lnPHE is the private health expenditures, lnCOE represents the natural log of carbon dioxide emissions, lnGDP shows the natural log of economic growth, lnFDI is the log of foreign direct investment, lnPAG represents the log of population aging, and lnEDU shows the log of education.

**Table 4:** Second generation unit root tests

Variables	CIPS		CADF	
	Levels	First Difference	Levels	First Difference
GHE	−1.542	−2.846 ***	−2.081	−4.105 ***
PHE	−1.872	−2.802 ***	−1.363	−2.844 **
COE	−1.469	−3.337 ***	−1.583	−3.642 ***
ENVI	−1.639	−4.146 ***	−1.469	−2.775 **
GDP	−1.080	−5.868 ***	−1.484	−2.648 **
FDI	−1.061	−3.541 ***	−1.856	−3.627 ***
PAG	−1.340	−2.913 ***	−2.136	−3.904 ***
EDU	−0.757	−5.011 ***	−1.998	−2.946 **

Note: ** statistical significance at 5% level and *** statistical significance at 1% level. The constants and trends are included in the Pesaran [73] unit root tests. The null hypothesis is rejected, it means that at least one country among all considered countries has stationarity and rejected null hypothesis is denoted by ***. The reported results are analyzed at lag = 1. The Pesaran [73] CIPS test critical values are obtained as −2.58 at 10%, −2.66 at 5%, and −2.81 at 1%, respectively. The critical values for CADF are as −2.810 at 1%, −2.66 at 5%, −2.580 at 1%, level of significance, respectively.

**Table 5:** LM Bootstrap Co-integration Test Results

Models	LM Statistic	Bootstrap *p*-Value
Model 1	22.480	0.970
Model 2	22.895	0.940
Model 3	23.125	0.912
Model 4	22.511	0.944

*Note*: The statistics in the bootstrap test are measured by using 5000 replications. This test is performed with null hypothesis that panel has cointegration against all units, whereas alternative hypothesis of panel has no cointegration.

**Table 6:** Panel Long Run Analysis.

Variables	Model 1		Model 2		Model 3		Model 4	
	GHE = f(CO_2_, GDP, FDI, PAG, EDU)		GHE = f(ENVI, GDP, FDI, PAG, EDU)		PHE = f(CO_2_, GDP, FDI, PAG, EDU)		PHE = f(ENVI, GDP, FDI, PAG, EDU)	
	CUP-FM	CUP-BC	CUP-FM	CUP-BC	CUP-FM	CUP-BC	CUP-FM	CUP-BC
CO_2_	0.10249 *** (4.0745)	0.15708 *** (7.1688)	-	-	−0.16020 *** (7.4875)	−0.05691 ** (2.7380)	-	-
ENVI	-	-	0.04506 *** (6.3886)	0.07437 *** (11.1053)	-	-	−0.02279 ** (2.7195)	-0.02301 ** (2.8774)
GDP	1.73879 *** (47.1776)	1.30172 *** (40.0070)	1.55056 *** (46.9651)	1.17807 *** (38.7515)	1.13731 *** (38.4690)	1.00734 *** (33.8487)	1.22973 *** (39.9744)	1.02558 *** (35.3202)
FDI	0.00015 (0.0745)	−0.01037 *** (−5.3408)	−0.00753 *** (−3.7935)	−0.00784 *** (−4.1886)	0.02587 *** (12.7556)	0.01354 *** (7.0058)	0.02355 *** (11.6094)	0.01286 *** (6.5835)
PAG	2.57254 *** (32.1706)	2.82168 *** (35.6509)	2.83083 *** (36.6002)	3.06076 *** (39.0916)	1.69591 *** (20.8132)	2.22361 *** (23.2364)	1.50994 *** (17.9205)	2.27830 *** (23.3924)
EDU	0.11388 *** (4.0727)	0.30233 *** (12.2282)	0.20731 *** (8.2875)	0.40414 *** (16.6107)	−0.29897 *** (−9.7285)	−0.47619 *** (−14.4527)	−0.47609 *** (−14.3028)	−0.46598 *** (−14.2501)

Note: ** statistical significance at 5% level and *** statistical significance at 1% level. In parentheses the values of t-statistics are given. The common factors’ numbers of continuously updated-fully modified (CUP-FM) and continuously updated- bias corrected (CUP-BC) techniques are determined through method of ICρ2 information. The SIC criterion based on PMG approach is used for the selection of optimum lag length.

**Table 7:** Dumitrescu and Hurlin heterogeneous panel causality test results.

Variables	lnGHE	lnPHE	lnCOE	lnENVI	lnGDP	lnFDI	lnPAG	lnEDU
lnGHE	-	3.9391 *** (0.000)	2.5282 ** (0.011)	3.5780 *** (0.000)	0.4405 (0.659)	4.1575 *** (0.000)	3.5507 *** (0.000)	0.6471 (0.517)
lnPHE	1.0375 (0.299)	-	3.4466 *** (0.000)	2.7000 *** (0.006)	−0.4218 (0.673)	6.4640 *** (0.000)	2.9485 *** (0.003)	0.0978 (0.922)
lnCOE	1.2440 (0.213)	0.4945 (0.620)	-	−0.1401 (0.888)	−0.0419 (0.966)	0.7378 (0.460)	0.9499 (0.342)	−0.5505 (0.582)
lnENVI	0.4776 (0.632)	0.5549 (0.579)	−0.1073 (0.914)	-	−0.1932 (0.846)	1.5958 (0.110)	2.6972 *** (0.007)	−0.3010 (0.763)
lnGDP	5.3802 *** (0.000)	1.9443 * (0.051)	2.2922 ** (0.021)	2.1043** (0.035)	-	2.9868 *** (0.002)	0.7081 (0.478)	0.3099 (0.756)
lnFDI	0.1850 (0.853)	0.3217 (0.747)	−0.4351 (0.663)	−0.1639 (0.869)	0.1228 (0.902)	-	3.7589 *** (0.000)	0.1170 (0.906)
lnPAG	2.2253 ** (0.026)	0.8552 (0.392)	2.0152 ** (0.043)	1.6244 (0.104)	1.3789 (0.167)	2.6776 *** (0.007)	-	3.5958 *** (0.000)
lnEDU	5.5806 *** (0.000)	1.8551 ** (0.063)	4.52340 *** (0.000)	3.3310 *** (0.000)	1.9284 * (0.053)	2.2257 ** (0.026)	6.0386 *** (0.000)	-

Note: * Statistical significance at 10% level; ** statistical significance at 5% level; *** statistical significance at 1% level. In parentheses p-values are given. The SIC criterion was used for the selection of optimum lag length.
